# βcatenin is a marker of poor clinical characteristics and suppressed immune infiltration in testicular germ cell tumors

**DOI:** 10.1186/s12885-018-4929-x

**Published:** 2018-11-03

**Authors:** Michal Chovanec, Zuzana Cierna, Viera Miskovska, Katarina Machalekova, Katarina Kalavska, Katarina Rejlekova, Daniela Svetlovska, Dusan Macak, Stanislav Spanik, Karol Kajo, Pavel Babal, Michal Mego, Jozef Mardiak

**Affiliations:** 1 2nd Department of Oncology, Faculty of Medicine, Comenius University and National Cancer Institute, Klenova 1, 833 10 Bratislava, Slovak Republic; 2 National Cancer Institute, Klenova 1, 833 10 Bratislava, Slovakia; 3 Department of Pathology, Faculty of Medicine, Comenius University, Sasinkova 4, 811 08 Bratislava, Slovakia; 4 1st Department of Oncology, Faculty of Medicine, Comenius University, Kollarska 12, 812 50 Bratislava, Slovakia; 5 St. Elisabeth Cancer Institute, Heydukova 10, 812 50 Bratislava, Slovakia; 6 Translational Research Unit, Faculty of Medicine, Comenius University, Klenova 1, 833 10 Bratislava, Slovakia; 7 Cancer Research Institute, Slovak Academy of Sciences, Dubravska cesta 9, 845 05 Bratislava, Slovakia; 8 Faculty Hospital with Policlinics Skalica, a.s, Koreszkova 936/7, 909 01 Skalica, Slovakia

**Keywords:** βcatenin, WNTβ pathway, PD-L1, Systemic-immune inflammation, Tumor infiltrating lymphocytes

## Abstract

**Background:**

WNT/βcatenin (WNTβ) pathway is activated in early stages of embryonic development. We aimed to evaluate the significance of βcatenin in germ cell tumors (GCTs) and explore associations with the inflamed environment.

**Methods:**

Surgical specimens from 247 patients were analyzed. Βcatenin expression was detected in the tumor tissue by immunohistochemistry and correlated with clinical characteristics, outcome, PD-L1 expression and systemic immune-inflammation index (SII). The Ingenuity Pathway Analysis (IPA) was used to investigate the immune-cell related effects of βcatenin and PD-L1 encoding genes.

**Results:**

βcatenin was expressed in 86.2% of GCTs. The expression in seminomas was significantly lower compared to all subtypes of non-seminoma (all *P* <  0.0001). A high expression (weighted histoscore > 150) was associated with primary mediastinal non-seminoma (*P* = 0.035), intermediate/poor risk disease (*P =* 0.033) and high tumor markers (*P =* 0.035). We observed a positive correlation with the PD-L1 in tumor and an inverse correlation with the SII. IPA uncovered relationships of *CTNNB* (βcatenin) and *CD274* (PD-L1) genes and their effects on differentiation, proliferation and activation of lymphocyte subtypes.

**Conclusion:**

Herein, we showed that βcatenin is associated with male adult GCT characteristics as well as supressed immune environment.

**Electronic supplementary material:**

The online version of this article (10.1186/s12885-018-4929-x) contains supplementary material, which is available to authorized users.

## Background

Testicular-germ cell tumors (GCTs) are chemotherapy sensitive malignancies [1, 2]. Little was known regarding immune mechanisms in this disease until recently. The significance of PD-1/PD-L1 pathway as well as the role of systemic-immune inflammation was previously described in our works [3, 4]. Additionally, changes in cytokine signalling was observed by our team in association with poor prognosis of GCTs [5, 6]. The role of immune check-point inhibition with anti-PD-1/anti-PD-L1/anti-CTLA4 agents has been established in numerous malignancies [7–10]. Data suggest that a T-cell inflamed microenvironment seems to be a predisposing factor for the efficacy of check-point inhibition covering only a subset of patients who benefit greatly from modern immune therapy [11, 12]. An activation of WNT/βcatenin (WNTβ) signalling pathway has been recently suggested as an intrinsic inhibitory mechanism for tumor T-cell infiltration in malignant melanoma pre-clinical model [13]. WNTβ pathway is known to be activated in early stages of embryonic development and it is involved in the regulation of differentiation of pluripotent cells [14, 15]. It has also been associated with carcinogenesis [16], cell proliferation and migration and the process of epithelial-mesenchymal transition [17]. Mutations in WNTβ have been identified in malignancies, such as hepatocellular, breast, colorectal and other cancer [18], however, the constitutive receptor stimulation can account for hyperactive WNTβ signalling in the absence of activating mutations as well [19]. Potapov et al. described the abundance of βcatenin in embryonal carcinoma identified by immunohistochemistry in 39 cases of testicular embryonal carcinomas [20]. Two studies reported differential expression in seminomas versus non-seminomas, but no correlation to resistance to chemotherapy was found [21, 22]. However, the role of βcatenin and WNTB signalling and its clinical implications in GCTs have not been comprehensively explored. In this retrospective study, we aimed to evaluate the role of βcatenin in GCTs and find correlations with systemic immune-inflammation and PD-L1 expression in tumor and tumor infiltrating lymphocytes (TILs).

## Methods

### Study patients

This translational study (chair M. Mego) included 247 patients with GCTs identified from the Slovak National Cancer Institute database, treated from January 1999 to December 2013 with available paraffin embedded tumor tissue specimen and sufficient follow-up clinical data. Patients with concurrent malignancy other than non-melanoma skin cancer in the previous 5 years were excluded. In all patients, data regarding age, tumor histology, clinical stage, type and number of metastatic sites, and delivery of systemic therapy were recorded and compared with βcatenin expression. Previously published data regarding PD-L1 expression on tumor/TILs and systemic immune-inflammation index (SII) in this cohort were available from 240 and 181 study patients, respectively [4, 23]. The Institutional Review Board (Ethics committee of Slovak National Cancer Institute in Bratislava approved this retrospective study (version 6.1 from 15 February 2017; ref. IZLO1) and a waiver of consent form patients was granted.

### Tumor pathology

Pathology review was conducted at the Department of Pathology, Faculty of Medicine, Comenius University, by two pathologists (ZC and PB) associated with the study.

### Diagnosis and tissue samples

Tumor tissue and normal testicular tissue were evaluated in all cases, when available. The study included tumor specimens from 247 patients before administration of systemic therapy. All but 9 of these specimens were obtained from primary testicular tumors. Biopsies of abdominal and mediastinal masses were performed in 7 and in 2 cases of primary extragonadal tumors, respectively. The GCTs were classified according to World Health Organization criteria [24]. Normal testicular tissue from non-cancer patients was not available for analysis, therefore we used normal tissue adjacent to testicular tumor for βcatenin expression evaluation, as described in previous studies [25, 26].

### Tissue microarray construction

According to the tumor histology, one or two representative tumor areas from each histological subtype (1–6 cores from each tumor) of germ cell tumor were identified on H&E sections. In case of mixed germ cell tumors, selected GCT histologies were sampled to isolate a specific histological pattern. Samples from normal testicular tissue were also marked, if present. Sections were matched to their corresponding wax blocks (the donor blocks), and 3-mm diameter cores of the tissues were removed from these donor blocks with the multipurpose sampling tool Harris Uni-Core (Sigma-Aldrich, Steinheim, Germany) and inserted into the recipient master block. The recipient block was cut into 5-μm sections and sections were transferred to coated slides. Tissue microarray construction and immunohistochemical staining was described in detail previously [4].

### Immunohistochemical staining

Slides were deparaffinized and rehydrated in phosphate buffered saline solution (10 mM, pH 7.2). The tissue epitopes were demasked using the automated water bath heating process in Dako PT Link (Dako, Glostrup, Denmark); the slides were incubated in TRIS-EDTA retrieval solution (10 mM TRIS, 1 mM EDTA pH 9.0) at 98 °C for 20 min. The slides were subsequently incubated for 1 h at room temperature with the primary mouse monoclonal antibody against Beta-Catenin (Dako, β-Catenin-1, IR702, Ready-to-Use) and immunostained using anti-mouse/anti-rabbit immuno-peroxidase polymer (EnVision FLEX/HRP, Dako, Glostrup, Denmark) for 30 min at room temperature, according to the manufacturer’s instructions. For visualization, the slides were reacted with diaminobenzidine substrate-chromogen solution (DAB, Dako, Glostrup, Denmark) for 5 min. Finally, the slides were counterstained with haematoxylin. Βcatenin positivity of epithelial cells in the colon was used as a positive control, same tissue with omitting of the primary antibody served as negative control.

### Immunohistochemical stain scoring

Tumor cores were independently assessed by two observers (ZC and PB) who were blinded to the clinicopathological data. In cases of disagreement, the consensus was made. Tumor cells with βcatenin expression were scored by a weighted histoscore (HS) which accounts for both the extent of cell staining and the staining intensity [27]. The portion of positive cells was estimated on a scale from 0 to 100%. The average intensity of positively staining cells was given a score from 0 to 3 (0 = no staining; 1 = weak; 2 = intermediate; and 3 = strong staining). The HS was then calculated by multiplying the percentage score by the intensity score, to yield a minimum value of 0 and a maximum value of 300. Based on the HS, a βcatenin expression was graded as low (0–150) or high (160–300) as we described previously [25]. If multiple histologic subtypes were present in a sample, we chose the highest number among these for a final βcatenin expression of a mixed GCT.

### Systemic immune-inflammation index

The SII is an index based on platelets (P), neutrophils (N) and lymphocytes (L) counts from the complete blood count. It was calculated as: SII = P x N/L as defined previously [28]. The median value (1003) obtained and validated in our previous study [23] was used as the cut-off value of SII, which was then dichotomized into low (below median) and high (above median) categories.

### Ingenuity pathway analysis (IPA)

We used IPA to further explore the immune related effects and interactions of *CTNNB1* (the gene encoding βcatenin) and *CD274* (the gene encoding PD-L1). *CTNNB1* and *CD274* genes were entered into IPA search engine. Immune related effects were subsequently selected and interactions between CTNNB1, CD274 and the selected effects were assessed to create the network of interactions.

### Statistical analysis

Patients’ characteristics were tabulated. A distribution of βcatenin HS was significantly different from the normal distribution (Shapiro–Wilk test), therefore we used non-parametric tests for analyses. Analyses of differences in distributions of βcatenin expression between the two groups of patients were performed using the Mann–Whitney U test, while Fisher’s exact test or the χ2 test when appropriate were used, when βcatenin expression was categorized as ‘low’ or ‘high’. A one-way analysis of variance and a Chi-square test were used for analyses of associations between the βcatenin and SII or PD-L1 expression.

A median follow-up period was calculated as a median observation time among all patients and among those still alive at the time of their last follow-up. PFS was calculated from the date of orchiectomy or the date of tumor biopsy to the date of progression or death or the date of the last adequate follow-up. OS was calculated from the date of orchiectomy or the date of tumor biopsy to the date of death or last the follow-up. PFS and OS were estimated using the Kaplan–Meier product limit method and compared by the log-rank test. All reported *P* values were two sided. A *P* value < 0.05 was considered as significant. Statistical analyses were performed using NCSS 2007 software (Hintze J, 2007, Kaysville, Utah, USA).

## Results

Patients’ characteristics are shown in Table 1**.** Majority of patients had a non-seminoma histology. A testicular tumor was the most common primary and more than two thirds of patients were within good risk according to the IGCCCG (International Germ Cell Cancer Collaborative Group). Tumor specimens from 247 patients before administration of systemic therapy included 50 pure seminomas, 128 non-seminomas (86 embryonal carcinomas, 19 yolk sac tumors, 1 choriocarcinoma, 22 teratomas) and 69 mixed GCTs. (Additional file 1: Table S1). Eight cases of seminomas were clinically considered as non-seminomas based on the positivity of alpha-fetoprotein.

**Table 1:** Patients’ characteristics

	*N* = 247	%
Age (years)		
Median (range)	30 (17–67)	
Histology		
Pure seminoma	50	20.2
Non-seminoma or mixed GCT	197	79.8
Primary tumor		
Gonadal	238	96.4
Primary retroperitoneal	7	2.8
Primary mediastinal	2	0.9
IGCCCG risk group		
Good risk	184	74.5
Intermediate risk	29	11.7
Poor risk	34	13.8
Sites of metastases		
Retroperitoneum	175	70.9
Mediastinum	32	13.0
Lungs	60	24.3
Liver	18	7.3
Other	34	13.8
Non-pulmonary visceral metastases	22	8.9
No. of metastatic sites		
0	63	25.5
1–2	141	57.1
> 3	43	17.4
Mean (range) of pre-treatment markers		
AFP mIU/ml	1274 (0–60,570)	
β-HCG IU/ml	10,412 (0–423,338)	
LDH (mkat/l)	12 (2–89)	

### Distribution of the βcatenin expression among histological subtypes

βcatenin expression was found in specimens from 213 of 247 patients (86.2%) in this cohort. We observed membranous staining only, cytoplasmic or nuclear staining was not present. Significantly more non-seminomas/mixed GCTs (97.3%) showed positive staining for βcatenin compared to seminomas (51.8%), (*P* <  0.0001, Fisher’s exact test). The expression in all seminomas (including pure seminomas and components in mixed tumors) was low or none (median = 5; interquartile range 50) while non-seminomas have generally shown a stronger expression (median = 200; interquartile range 100), (*P* <  0.0001). Detailed expression in all subtypes is shown in Table 2 and Additional file 2: Figure S1.

**Table 2:** βcatenin expression in different histologic subtypes of germ cell tumors

Histologic subtype			Expression of βcatenin						
	N	Median	Interquartile range	*p*-value	Low		High		*p*-value
					N	%	N	%	
Seminoma	83	5	50	NA	81	97.6	2	2.4	NA
GCNIS	73	200	100	< 0.001	35	48.0	38	52.9	< 0.001
Embryonal carcinoma	134	200	100	< 0.001	63	47.0	71	53.0	< 0.001
Yolk sac tumor	31	200	0	< 0.001	7	22.6	24	77.4	< 0.001
Choriocarcinoma	13	100	165	< 0.001	7	53.8	6	46.2	< 0.001
Teratoma	58	100	173	< 0.001	32	55.2	26	44.8	< 0.001

**Fig. 1:**
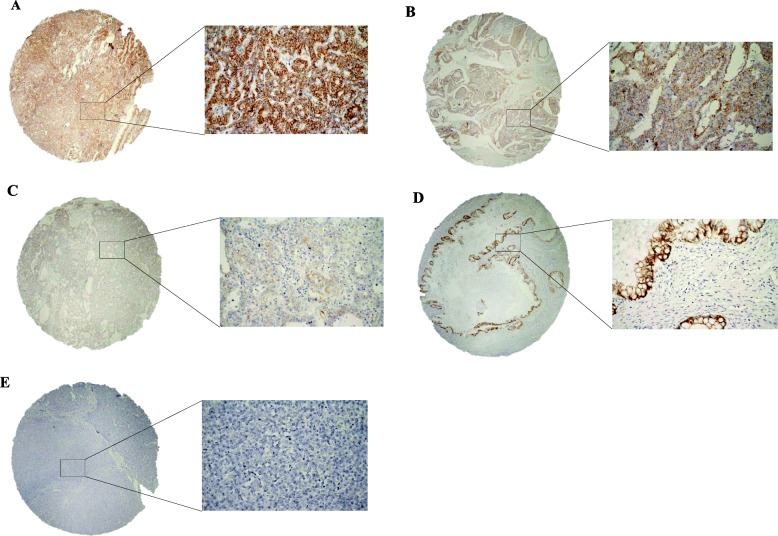
Immunohistochemical detection of βcatenin expression in testicular germ cell tumours. **a** Yolk sac tumour showed constant strong membranous βcatenin positivity (brown colour); **b** Embryonal carcinoma with constant moderate membranous βcatenin positivity; **c** Embryonal carcinoma with focal week membranous βcatenin positivity; **d** Teratoma with constant strong membranous βcatenin positivity in epithelial cells and negativity (blue colour) in mesenchymal cells. **e** Seminoma βcatenin negative. Original magnification ×40/× 400

All histological subtypes of non-seminoma showed any intensity in staining more frequently than seminomas (embryonal carcinoma 98.5%, teratoma 81.1%, yolk sac tumor 96.8%, choriocarcinoma 76.9%), (*P* <  0.0001 for EC, T, YST and *P* = 0.074 for CHC), (Fig. 1). Proportion of patients with high βcatenin expression (HS > 150) was significantly higher in GCNIS, YST and EC and lower in seminoma compared to adjacent normal testicular tissue (Table 2**)**.

### βcatenin and patients’ characteristics

βcatenin has shown significant associations with several clinical characteristics of patients in this cohort. Patients with testicular and retroperitoneal primary GCT had lower expression of βcatenin compared to patients with primary mediastinal non-seminoma (PMNSGCT) (*P =* 0.035) **(**Table 3). βcatenin was higher among intermediate and poor IGCCCG risk groups as opposed to good risk patients (*P =* 0.033). Similarly, the higher expression was seen in patients with highly elevated tumor markers (S2–3) compared to patients with no or mild marker elevation (S0–1) (*P =* 0.035). Metastatic sites were generally not associated with differences in the βcatenin expression (Table 3).

**Table 3:** Patients’ characteristics according to the expression of βcatenin

Variable			βcatenin						
	N	Median	interquartile range	*p*-value	Low		High	*p*-value	
					N	%	N	%	
All patients	247	100	150	NA	131	53	116	47	
Clinical subtype									
Pure seminoma	50	50	100	< 0.0001	42	84	8	16	< 0.0001
Non-seminoma	197	200	115		89	45	108	55	
Tumor primary									
Testicular/retroperitoneal	245	100	150	0.035	131	53	114	47	0.131
Mediastinal	2	300	N/A		0	0	2	100	
IGCCCG risk group									
Good	184	100	170	0.033	105	57	79	43	0.030
Intermediate/Poor	63	200	130		26	42	37	58	
Number of metastatic sites									
0	63	100	100	0.408	32	51	31	49	0.813
1 to 2	142	100	175		75	53	67	47	
≥ 3	42	100	142.5		24	57	18	43	
Retroperitoneal LN metastases									
Absent	72	130	100	0.075	36	50	36	50	0.540
Present	175	100	175		95	54	80	46	
Mediastinal LN metastases									
Absent	215	100	150	0.598	117	54	98	46	0.260
Present	32	200	168.75		14	44	18	56	
Lung metastases									
Absent	187	100	150	0.967	98	52	89	48	0.726
Present	60	100	145		33	55	27	45	
Liver									
Absent	229	100	150	0.243	124	54	105	46	0.212
Present	18	200	107.5		7	39	11	61	
Non-pulmonary visceral metastases									
Absent	225	100	150	0.891	120	53	105	47	0.765
Present	22	150	150		11	50	11	50	
S – stage									
0–1	187	100	170	0.035	107	57	80	43	0.020
2–3	60	200	130		24	40	36	60	

### βcatenin and patient outcome

A survival analysis did not show differences among patients expressing high versus low amounts of βcatenin prior to treatment for PFS and OS; (HR 0.70, 95% CI 0.41–1.19, *P* = 0.185) and (HR 0.89, 95% CI 0.45–1.74, *P* = 0.727), respectively **(**Additional file 3: Figure S2). Sub-analyses for survival in patients with seminoma and non-seminoma also did not show statistical differences for PFS and OS (data not shown). In the subsequent analyses, we assessed the βcatenin and its’ correlations with PD-L1 expression and the SII, which were previously assessed in the same patient cohort [3, 4, 23].

### The association with PD-L1 on tumor cells, TILs and SII

The expression of PD-L1 on tumor cells have shown a significant correlation with the expression of βcatenin. The expression of PD-L1 was originally reported in our previous studies [3, 4]. Samples from 240 patients were previously examined for PD-L1 and currently for the expression of βcatenin. Patients with low βcatenin had lower expression of PD-L1 on tumor cells compared to patients with the high βcatenin (median = 40 vs 67.5, respectively; *P* = 0.010). Spearman’s correlation analysis confirmed the significant correlation between PD-L1 on tumor cells and βcatenin (*r* = 0.316; *P* = 0.0002). The PD-L1 expression on TILs did not significantly differ among patients with low (median = 100) vs high (median = 60) expression of βcatenin (*P* = 0.220).

βcatenin was significantly higher in patients with low SII (median = 200) vs high SII (median = 100) (*P* = 0.011).

### βcatenin and the infiltration with TILs

We have performed an analysis to explore the association of βcatenin and the presence of TILs in tumor specimen of patients from our previous study [4]. Samples from 240 patients were previously examined for TILs and currently for the expression of βcatenin. Of 240 evaluated patients, 225 (93.7%) had their tumor infiltrated with TILs. In our cohort, patients with no TILs did not have significantly higher expression of βcatenin compared to patients with TILs present in the specimen (median = 200 vs 100, respectively; *P* = 0.083).

### IPA

**Fig. 2:**
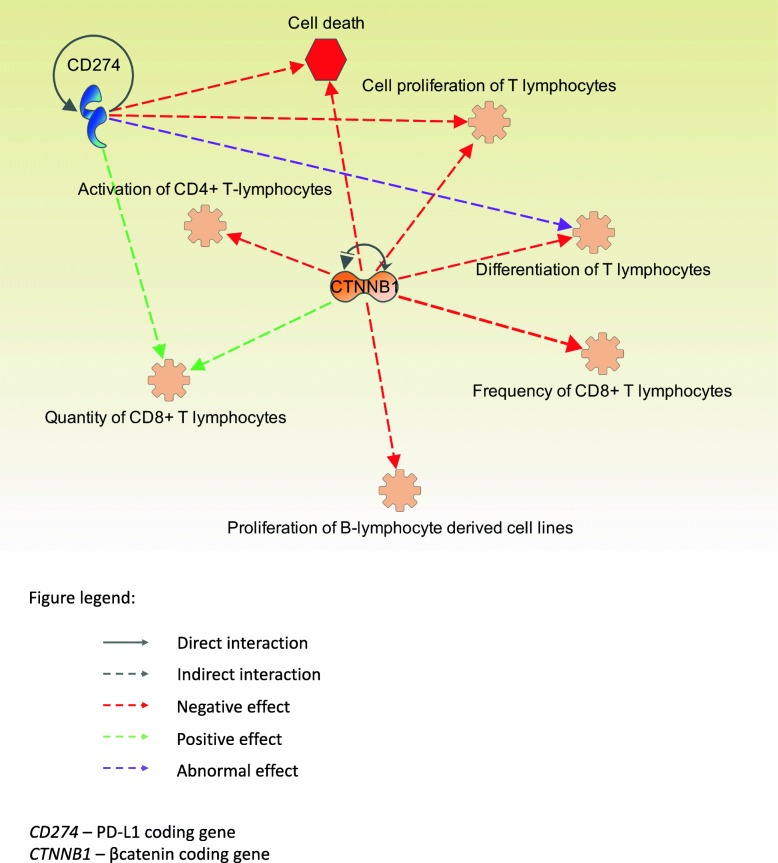
The ingenuity pathway analysis of immune-related effects of *CTNNB1* and *CD274. CD274* to cell death: active *CD274* decreases apoptosis of cells. Inhibition of active *CD274* increases apoptosis of cells. *CD274* to cell proliferation of T lymphocytes: active *CD274* decreases proliferation of T cells. *CD274* to differentiation od T lymphocytes: mutant *CD274* is involved in abnormal differentiation of T cells. *CD274* to quantity of CD8+ T lymphocytes: mutant *CD274* is increases number of CD8+ T cells. *CTNNB1* to cell death: human *CTNNB1* decreases death of cells. *CTNNB1* to differentiation of T lymphocytes: active *CTNNB1* increases the arrest in growth of thymocytes (thymic progenitors of T-cells). *CTNNB1* to frequency of CD8+ T lymphocytes: active mutant *CTNNB1* decreases differentiation thymocytes expressing CD4 and CD8 proteins. *CTNNB1* to proliferation of B-lymphocyte derived cell lines: interference of *CTNNB1* mRNA by siRNA decreases proliferation of pre-B cells (PD36 cells). *CTNNB1* to quantity of CD8+ lymphocytes: activated mutant CTNNB1 in dendritic cells abnormally increases the number of CD8+ T cells in mouse spleen that are infected with vaccinia virus or Toxoplasma gondii. *CTNNB1* to activation of CD4+ T lymphocytes: degradation of CTNNB1 protein is involved in activation of CD4+ T cells

We have discovered several effects of aberrant CTNNB1 and CD274 (upregulations, mutations or interactions) related to differentiation, proliferation and activation of CD4+ and CD8+ lymphocytes. The results of the IPA are summarized in Fig. 2.

## Discussion

In this translational study, we assessed the significance of the βcatenin expression in patients with GCT prior to administration of chemotherapy. We performed a survival analysis and correlated its’ expression to clinical and pathological characteristics. We identified a significant difference in the expression of βcatenin between seminomas and non-seminomas. A study by Fritsch et al. reported activated WNTβ and differential βcatenin expression in female paediatric GCT patients [29]. All non-seminoma subtypes in our study have shown strong staining for βcatenin with the highest expressions seen in embryonal carcinoma and yolk sac tumor. This finding can be attributed to the pluripotency of embryonal cancer cells that are known to be malignant counterparts of embryonic stem cells and act as stem cells in the development of GCTs. However, the implication for yolk sac tumor is unclear [30, 31]. Raggioli et al. demonstrated that βcatenin was vital for the maintenance of pluripotency associated genes in mouse embryonic stem cell lines. In addition, removal of βcatenin led to a strong increase of cell death [32]. Our study suggests the loss of βcatenin during the development from the progenitor cell, as only a weak or no expression was seen in seminomas. We hypothesize it can be responsible for different biology and treatment sensitivity compared to non-seminoma GCTs. The loss of other markers of pluripotency, such as SOX2 and CD30 was well described in the past [33]. Our further analysis uncovered differences in βcatenin expression in association with poor-risk clinical features. Primary testicular and retroperitoneal tumors had lower expression of βcatenin in comparison with PMNSGCTs which represent the worst prognostic group of GCT patients [34]. However, this sub-analysis is underpowered due to small number of PMNSGCTs in the cohort. The underlying connection to βcatenin and WTNβ signalling suggest that patients with tumors that have higher abilities for pluripotency belong to poorer risk category and produce more tumor markers. Although βcatenin assessed in our cohort of chemotherapy naïve patients did not show a prognostic significance in terms of PFS and OS, further studies assessing the βcatenin in relapsed or platinum refractory patients are needed to explore its’ clinical significance.

Our subsequent analysis evaluated βcatenin and its possible associations with the tumor immune microenvironment. Our previous works have shown prognostic significance of PD-L1 expressed on tumor cells and TILs and systemic inflammation in GCTs [3, 4, 23]. Herein we identified the positive correlation of βcatenin to PD-L1 expression on tumor cells, but not on TILs. High PD-L1 expression on TILs was linked to a better outcome, whereas high PD-L1 expression on tumor cells was predictive of poor outcome in GCTs. The role of βcatenin and its’ interaction with PD-1/PD-L1 pathway is not clear. Its’ association with PD-L1 on tumor cells but not on TILs may suggest the independence of PD-L1 positive TILs from WNTβ signalling. Moreover, the association with immune inflamed microenvironment was confirmed in inverse correlation with SII suggesting that patients with low inflammatory activity had activated WNTβ signalling. Spranger et al. observed a lack of T-cell infiltrate in autochtonous mouse models, which led to resistance to anti-PD-L1/anti-CTLA4 monoclonal antibody therapy. Subsequently, authors were able to restore T-cell infiltration in mice by introduction of injected dendritic cells harvested from transgenic mice [13]. We did not observe a higher expression of βcatenin in patients without TILs in tumor specimens, perhaps due to insufficient size of our cohort. Our subsequent ingenuity pathway analysis uncovered important roles of *CTNNB1* and *CD274* genes in the process of differentiation, proliferation and function of CD8+ and CD4+ T cells. The increase in number of CD8+ positive cells resulting from aberrant mutated *CTNNB1* is seemingly conflicting with other described effects of WNT signalling, however, Cohen et al. described that these cells failed to be activated due to a lack of cross-presentation resulting from increased splenic CD8α dendritic cell activity [35]. Furthermore, animal model experiments performed by Augustin et al. in mouse embryonic stem cells with overexpressed WNT secretion factor Evi/WIs, showed an increased tumor growth and impaired immune cell recruitment in the presence of enhanced Evi expression. Authors observed reduced T-cell and B-cell infiltration especially in Evi overexpressing teratomas, thus, showing the negative effect of WNT signalling on immune surveillance [36].

Our study has some strengths and limitations. The large patient population creates a relatively balanced population for the biomarker assessment. Additionally, the availability of the PD-L1 expression and the SII data from the same set of patients creates a unique cohort with the possibility to uncover associations among these. Limitations include the retrospective nature of the analysis and underrepresentation of extragonadal germ cell tumors. The membranous expression of βcatenin does not provide clear evidence of WNT pathway signalling, thus the expression of a target gene would provide more information. Our selection of βcatenin to explore as a biomarker in this hypothesis generating study may be biased, therefore we plan a more comprehensive proteomic analysis in the future.

## Conclusion

In conclusion, this is the first translational study to assess the biological, prognostic and clinical significance of βcatenin in GCTs of adult men. We uncovered the differences between seminomas and non-seminomas. We have shown associations with several clinical characteristics and systemic immune-inflammation and PD-L1 on tumor cells, thus providing the insights into immunobiology of GCTs. More studies are needed to validate this exploratory trial and to explore the possible therapeutic implications.

## Additional files

Additional file 1: **Table S1. **Composition of mixed GCTs (*N* = 69). (DOCX 14 kb)

Additional file 2: **Figure S1.** Box-plot representation of difference in the expression of βcatenin between seminoma (median = 5, IQR = 50) and other histological subtypes (1); A - germ-cell neoplasia in situ (median = 200, IQR = 100); B – embryonal carcinoma (median = 200, IQR = 100); C – yolk sac tumor (median = 200, IQR = 0); D – choriocarcinoma (median = 100, IQR = 165); E – teratoma (median = 200, IQR = 173). (DOCX 37 kb)

Additional file 3: **Figure S2A.** Kaplan-Meier estimates of probabilities of progression-free survival according to the expression of βcatenin in patients with GCTs (*n* = 247), Hazard ratio 0.70, 95% CI 0.41–1.19), *P* = 0.185; low βcatenin histoscore < 150, high βcatenin histoscore > 150). **Figure S2B**. Kaplan-Meier estimates of probabilities of overall survival according to the expression of βcatenin in patients with GCTs (n = 247), Hazard ratio 0.89, 95% CI 0.45–1.74), *P* = 0.727; low βcatenin histoscore < 150, high βcatenin histoscore > 150). (DOCX 32 kb)

## Abbreviations

GCT: Germ cell tumor
H&E: Hematoxylin eosin
HS: Histoscore
IGCCCG: International Germ Cell Cancer Collaborative Group
IPA: Ingenuity pathway analysis
L: Lymphocytes
N: Neutrophils
OS: Overall survival
P: Platelets
PFS: Progression free survival
SII: Systemic immune-inflammation index
TIL: Tumor infiltrating lymphocytes
