# Supplementary material for: βcatenin is a marker of poor clinical characteristics and suppressed immune infiltration in testicular germ cell tumors
Source: BMC Cancer. 2018 Nov 3;18:1062. doi: 10.1186/s12885-018-4929-x (PMC6215644; doi:10.1186/s12885-018-4929-x)
Supplement: Supplementary file 1 — Table S1. Composition of mixed GCTs (N = 69). (DOCX 14 kb) [file 12885_2018_4929_MOESM1_ESM.docx]

**Table S1** Composition of mixed GCTs (*N* = 69)

| **Histological subtype** | | | | | **Number of patients** |
| --- | --- | --- | --- | --- | --- |
| EC | SEM |  |  |  | 11 |
| EC |  |  |  | TER | 34 |
| EC |  |  | ChC | TER | 1 |
| EC |  |  | ChC |  | 3 |
|  | SEM |  | ChC |  | 1 |
| EC | SEM |  | ChC |  | 3 |
|  | SEM |  |  | TER | 1 |
|  |  | YST |  | TER | 5 |
| EC | SEM |  |  | TER | 4 |
|  | SEM | YST |  |  | 2 |
| EC |  | YST | ChC | TER | 1 |
|  | SEM | YST |  | TER | 1 |
|  |  | YST | ChC |  | 2 |

**Abbreviations:** EC, embryonal carcinoma; SEM, seminoma; YST, yolk sac tumour; ChC, choriocarcinoma; TER, teratoma
