# Supplementary material for: βcatenin is a marker of poor clinical characteristics and suppressed immune infiltration in testicular germ cell tumors
Source: BMC Cancer. 2018 Nov 3;18:1062. doi: 10.1186/s12885-018-4929-x (PMC6215644; doi:10.1186/s12885-018-4929-x)
Supplement: Supplementary file 2 — Figure S1. Box-plot representation of difference in the expression of βcatenin between seminoma (median = 5, IQR = 50) and other histological subtypes (1); A - germ-cell neoplasia in situ (median = 200, IQR = 100); B – embryonal carcinoma (median = 200, IQR = 100); C – yolk sac tumor (median = 200, IQR = 0); D – choriocarcinoma (median = 100, IQR = 165); E – teratoma (median = 200, IQR = 173). (DOCX 37 kb) [file 12885_2018_4929_MOESM2_ESM.docx]

Figure S1. Box-plot representation of difference in the expression of βcatenin between seminoma (median = 5, IQR = 50) and other histological subtypes (1); A - germ-cell neoplasia in situ (median = 200, IQR = 100); B – embryonal carcinoma (median = 200, IQR = 100); C – yolk sac tumor (median = 200, IQR = 0); D – choriocarcinoma (median = 100, IQR = 165); E – teratoma (median = 200, IQR = 173)

Abbreviations: IQR – interquartile range

A

B

C

D

E
