# Supplementary material for: βcatenin is a marker of poor clinical characteristics and suppressed immune infiltration in testicular germ cell tumors
Source: BMC Cancer. 2018 Nov 3;18:1062. doi: 10.1186/s12885-018-4929-x (PMC6215644; doi:10.1186/s12885-018-4929-x)
Supplement: Supplementary file 3 — Figure S2A. Kaplan-Meier estimates of probabilities of progression-free survival according to the expression of βcatenin in patients with GCTs (n = 247), Hazard ratio 0.70, 95% CI 0.41–1.19), P = 0.185; low βcatenin histoscore < 150, high βcatenin histoscore > 150). Figure S2B. Kaplan-Meier estimates of probabilities of overall survival according to the expression of βcatenin in patients with GCTs (n = 247), Hazard ratio 0.89, 95% CI 0.45–1.74), P = 0.727; low βcatenin histoscore < 150, high βcatenin histoscore > 150). (DOCX 32 kb) [file 12885_2018_4929_MOESM3_ESM.docx]

Figure S2A. Kaplan-Meier estimates of probabilities of progression-free survival according to the expression of βcatenin in patients with GCTs (n = 247), Hazard ratio 0.70, 95% CI 0.41-1.19), P = 0.185; low βcatenin histoscore < 150, high βcatenin histoscore > 150)

Figure S2B. Kaplan-Meier estimates of probabilities of overall survival according to the expression of βcatenin in patients with GCTs (n = 247), Hazard ratio 0.89, 95% CI 0.45-1.74), P = 0.727; low βcatenin histoscore < 150, high βcatenin histoscore > 150)
